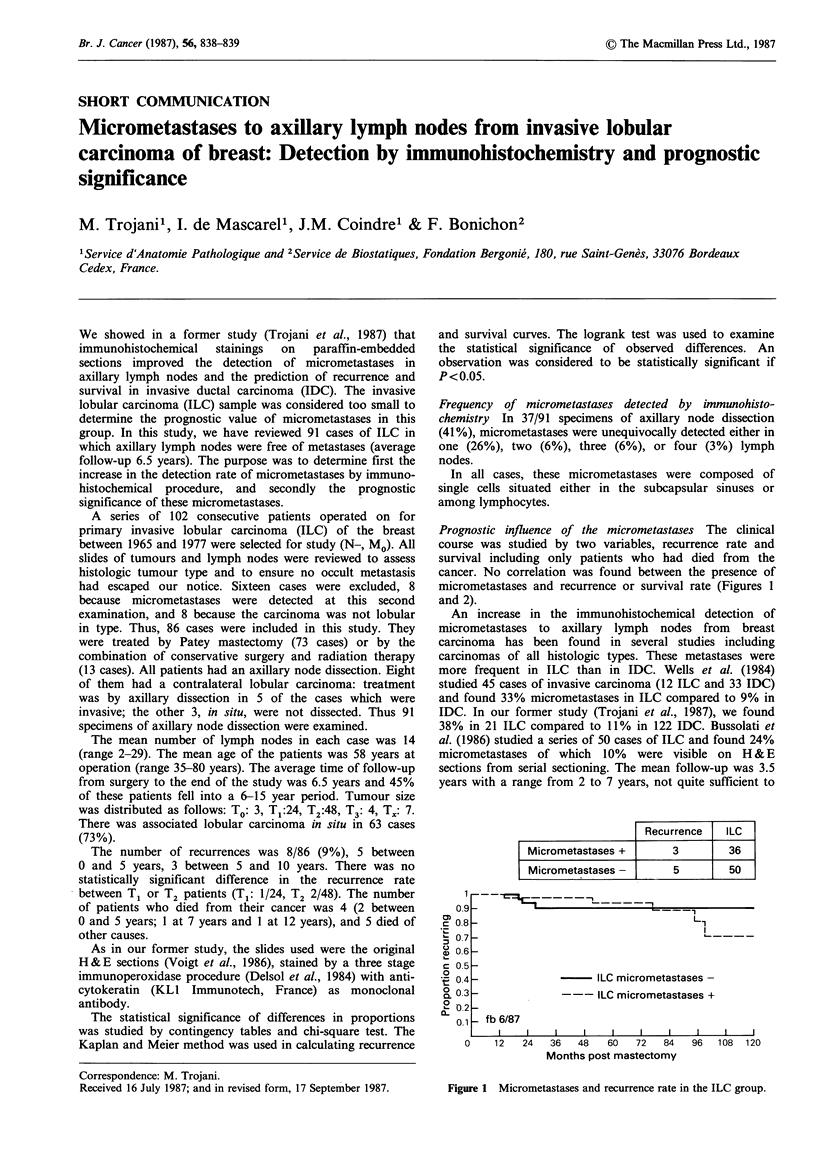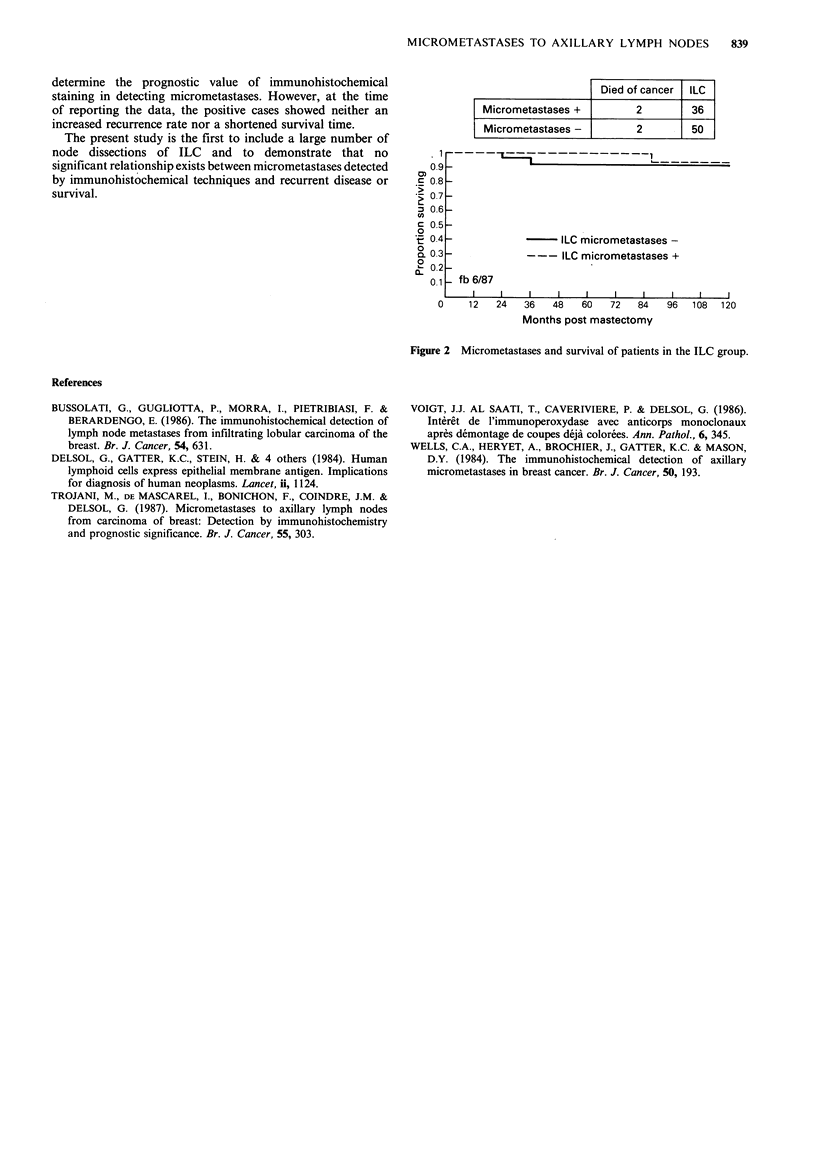# Micrometastases to axillary lymph nodes from invasive lobular carcinoma of breast: detection by immunohistochemistry and prognostic significance.

**DOI:** 10.1038/bjc.1987.301

**Published:** 1987-12

**Authors:** M. Trojani, I. de Mascarel, J. M. Coindre, F. Bonichon

**Affiliations:** Service d'Anatomie Pathologique, Fondation Bergonié, Bordeaux, France.


*Br. J. Cancer* (1987), **56**, 838–839

© The Macmillan Press Ltd., 1987

SHORT COMMUNICATION

### Micrometastases to axillary lymph nodes from invasive lobular carcinoma of breast: Detection by immunohistochemistry and prognostic significance

M. Trojani[1], I. de Mascarel[1], J.M. Coindre[1] & F. Bonichon[2]

[1]*Service d'Anatomie Pathologique and* [2]*Service de Biostatiques, Fondation Bergonié, 180, rue Saint-Genès, 33076 Bordeaux Cedex, France.*

We showed in a former study (Trojani *et al.*, 1987) that immunohistochemical stainings on paraffin-embedded sections improved the detection of micrometastases in axillary lymph nodes and the prediction of recurrence and survival in invasive ductal carcinoma (IDC). The invasive lobular carcinoma (ILC) sample was considered too small to determine the prognostic value of micrometastases in this group. In this study, we have reviewed 91 cases of ILC in which axillary lymph nodes were free of metastases (average follow-up 6.5 years). The purpose was to determine first the increase in the detection rate of micrometastases by immunohistochemical procedure, and secondly the prognostic significance of these micrometastases.

A series of 102 consecutive patients operated on for primary invasive lobular carcinoma (ILC) of the breast between 1965 and 1977 were selected for study (N–, $M_0$). All slides of tumours and lymph nodes were reviewed to assess histologic tumour type and to ensure no occult metastasis had escaped our notice. Sixteen cases were excluded, 8 because micrometastases were detected at this second examination, and 8 because the carcinoma was not lobular in type. Thus, 86 cases were included in this study. They were treated by Patey mastectomy (73 cases) or by the combination of conservative surgery and radiation therapy (13 cases). All patients had an axillary node dissection. Eight of them had a contralateral lobular carcinoma: treatment was by axillary dissection in 5 of the cases which were invasive; the other 3, *in situ*, were not dissected. Thus 91 specimens of axillary node dissection were examined.

The mean number of lymph nodes in each case was 14 (range 2–29). The mean age of the patients was 58 years at operation (range 35–80 years). The average time of follow-up from surgery to the end of the study was 6.5 years and 45% of these patients fell into a 6–15 year period. Tumour size was distributed as follows: $T_0$: 3, $T_1$:24, $T_2$:48, $T_3$: 4, $T_x$: 7. There was associated lobular carcinoma *in situ* in 63 cases (73%).

The number of recurrences was 8/86 (9%), 5 between 0 and 5 years, 3 between 5 and 10 years. There was no statistically significant difference in the recurrence rate between $T_1$ or $T_2$ patients ($T_1$: 1/24, $T_2$ 2/48). The number of patients who died from their cancer was 4 (2 between 0 and 5 years; 1 at 7 years and 1 at 12 years), and 5 died of other causes.

As in our former study, the slides used were the original H & E sections (Voigt *et al.*, 1986), stained by a three stage immunoperoxidase procedure (Delsol *et al.*, 1984) with anti-cytokeratin (KL1 Immunotech, France) as monoclonal antibody.

The statistical significance of differences in proportions was studied by contingency tables and chi-square test. The Kaplan and Meier method was used in calculating recurrence and survival curves. The logrank test was used to examine the statistical significance of observed differences. An observation was considered to be statistically significant if $P < 0.05$.

*Frequency of micrometastases detected by immunohistochemistry* In 37/91 specimens of axillary node dissection (41%), micrometastases were unequivocally detected either in one (26%), two (6%), three (6%), or four (3%) lymph nodes.

In all cases, these micrometastases were composed of single cells situated either in the subcapsular sinuses or among lymphocytes.

*Prognostic influence of the micrometastases* The clinical course was studied by two variables, recurrence rate and survival including only patients who had died from the cancer. No correlation was found between the presence of micrometastases and recurrence or survival rate (Figures 1 and 2).

An increase in the immunohistochemical detection of micrometastases to axillary lymph nodes from breast carcinoma has been found in several studies including carcinomas of all histologic types. These metastases were more frequent in ILC than in IDC. Wells *et al.* (1984) studied 45 cases of invasive carcinoma (12 ILC and 33 IDC) and found 33% micrometastases in ILC compared to 9% in IDC. In our former study (Trojani *et al.*, 1987), we found 38% in 21 ILC compared to 11% in 122 IDC. Bussolati *et al.* (1986) studied a series of 50 cases of ILC and found 24% micrometastases of which 10% were visible on H & E sections from serial sectioning. The mean follow-up was 3.5 years with a range from 2 to 7 years, not quite sufficient to

| | Recurrence | ILC |
|---|---|---|
| Micrometastases + | 3 | 36 |
| Micrometastases − | 5 | 50 |

**Figure 1** Micrometastases and recurrence rate in the ILC group.

Correspondence: M. Trojani.

Received 16 July 1987; and in revised form, 17 September 1987.

determine the prognostic value of immunohistochemical staining in detecting micrometastases. However, at the time of reporting the data, the positive cases showed neither an increased recurrence rate nor a shortened survival time.

The present study is the first to include a large number of node dissections of ILC and to demonstrate that no significant relationship exists between micrometastases detected by immunohistochemical techniques and recurrent disease or survival.

| | Died of cancer | ILC |
|---|---|---|
| Micrometastases + | 2 | 36 |
| Micrometastases − | 2 | 50 |

**Figure 2** Micrometastases and survival of patients in the ILC group.

### References

BUSSOLATI, G., GUGLIOTTA, P., MORRA, I., PIETRIBIASI, F. & BERARDENGO, E. (1986). The immunohistochemical detection of lymph node metastases from infiltrating lobular carcinoma of the breast. *Br. J. Cancer,* **54,** 631.

DELSOL, G., GATTER, K.C., STEIN, H. & 4 others (1984). Human lymphoid cells express epithelial membrane antigen. Implications for diagnosis of human neoplasms. *Lancet,* **ii,** 1124.

TROJANI, M., DE MASCAREL, I., BONICHON, F., COINDRE, J.M. & DELSOL, G. (1987). Micrometastases to axillary lymph nodes from carcinoma of breast: Detection by immunohistochemistry and prognostic significance. *Br. J. Cancer,* **55,** 303.

VOIGT, J.J. AL SAATI, T., CAVERIVIERE, P. & DELSOL, G. (1986). Intèrêt de l'immunoperoxydase avec anticorps monoclonaux après démontage de coupes déjà colorées. *Ann. Pathol.,* **6,** 345.

WELLS, C.A., HERYET, A., BROCHIER, J., GATTER, K.C. & MASON, D.Y. (1984). The immunohistochemical detection of axillary micrometastases in breast cancer. *Br. J. Cancer,* **50,** 193.